# Subcutaneous-implantable cardioverter defibrillator lead dislodgement in a juvenile catecholamine-induced polymorphic ventricular tachycardia patient

**DOI:** 10.1093/omcr/omac130

**Published:** 2022-12-16

**Authors:** Miwa Miyoshi, Hajime Saeki, Yo Arita, Yoshinori Iida, Tomoki Fukui, Shohei Yamamoto, Kana Shichijo, Yuto Suetani, Kosuke Hirose, Miho Kuramoto, Nobuyuki Ogasawara

**Affiliations:** Cardiovascular Division, Japan Community Health care Organization Osaka Hospital, Fukushima, Fukushima-ku Osaka Japan, Japan; Cardiovascular Division, Japan Community Health care Organization Osaka Hospital, Fukushima, Fukushima-ku Osaka Japan, Japan; Cardiovascular Division, Japan Community Health care Organization Osaka Hospital, Fukushima, Fukushima-ku Osaka Japan, Japan; Cardiovascular Division, Japan Community Health care Organization Osaka Hospital, Fukushima, Fukushima-ku Osaka Japan, Japan; Cardiovascular Division, Japan Community Health care Organization Osaka Hospital, Fukushima, Fukushima-ku Osaka Japan, Japan; Cardiovascular Division, Japan Community Health care Organization Osaka Hospital, Fukushima, Fukushima-ku Osaka Japan, Japan; Cardiovascular Division, Japan Community Health care Organization Osaka Hospital, Fukushima, Fukushima-ku Osaka Japan, Japan; Cardiovascular Division, Japan Community Health care Organization Osaka Hospital, Fukushima, Fukushima-ku Osaka Japan, Japan; Cardiovascular Division, Japan Community Health care Organization Osaka Hospital, Fukushima, Fukushima-ku Osaka Japan, Japan; Cardiovascular Division, Japan Community Health care Organization Osaka Hospital, Fukushima, Fukushima-ku Osaka Japan, Japan; Cardiovascular Division, Japan Community Health care Organization Osaka Hospital, Fukushima, Fukushima-ku Osaka Japan, Japan

## Abstract

Catecholaminergic polymorphic ventricular tachycardia (CPVT) is a relatively rare inherited arrhythmic disease that causes sudden cardiac death, and is caused by mutations in the cardiac ryanodine receptor (RyR2) or sarcoplasmic reticulum protein calsequestrin 2 gene (CASQ2). A 16-year-old man was diagnosed with CPVT and was implanted with a Subcutaneous-implantable Cardioverter Defibrillator (S-ICD), but defibrillation electrode detachment occurred early after placement. We suspected that a two-incision technique was the possible cause. We also report on changes in surface ECG in remote monitoring of the device.

**TAKE HOME MESSAGE**

Although two-incision techniques are becoming the mainstream method of S-ICD implantation, we should consider that the three-incision technique may be advantageous in highly active patients.

Remote monitoring may also be useful for early detection of S-ICD dislodgement.

## INTRODUCTION

Implantable cardioverter-defibrillator (ICD) is an established therapeutic device for the prevention of sudden cardiac death [1]. Subcutaneous-ICD (S-ICD) was listed in the 2015 European Society of Cardiology (ESC) guidelines [2] as an alternative therapeutic option with a class-IIa recommendation in patients for whom is indicated ICD not requiring pacing for bradycardia, cardiac resynchronization therapy or anti-tachycardia pacing. However, problems and complications associated with the device are relevant and may lead to a poor prognosis. Transvenous electrodes of ICDs have caused problems such as lead injury and infection, myocardial damage and pericarditis. S-ICD is a completely extra thoracic and extravascular system. Therefore, it can be removed even if infection of a pacemaker occurs. Little has been reported on lead dislodgement with S-ICD. We experienced lead dislodgement of S-ICD within 1 month following the two-incision technique in a patient with catecholaminergic polymorphic ventricular tachycardia (CPVT). CPVT is a relatively rare genetic arrhythmia disorder. Using the two-incision technique for lead fixation to active and sporting patients may induce lead dislodgement, and the three-incision technique may be preferable.

## CASE REPORT

**Figure 1:**
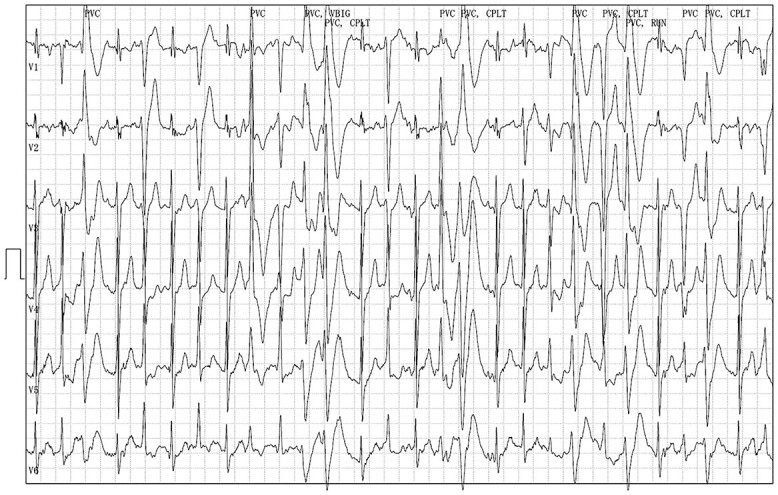
Twelve-lead ECG displayed in the treadmill test, and two types of ventricular arrhythmias appeared on the electrocardiogram during the exercise load, namely, the lower and the upper axis.

**Figure 2:**
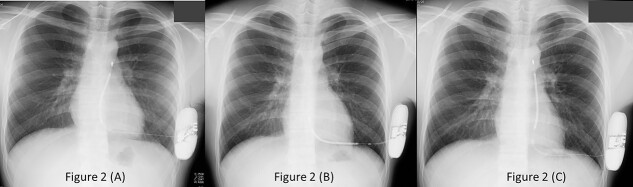
(A) Chest X-ray showing that the extra thoracic lead and generator of S-ICD were first located in the left lateral side; (B) outpatient chest X-ray showed dislodgement of the defibrillation electrode leads in the Para sternum; (C) chest X-ray after replacement of S-ICD using a three-incision technique.

A 16-year-old male visited our hospital complaining of fainting during swimming and exercise. He almost drowned during swimming and underwent cardiopulmonary resuscitation twice. He belonged to the baseball club and was a pitcher and batter. No special abnormalities were identified in the 12-lead Electrocardiogram (ECG) and echocardiography. Bidirectional ventricular tachycardia appeared frequently during a treadmill testing (Fig. 1). CPVT [3, 4] was suspected and so he was treated with flecainide and carvedilol. In addition, genetic testing at the National Cerebral and Cardiovascular Center (Suita, Japan) revealed a point mutation in exon8 of the ryanodine (RYR2) receptor. He was admitted at our hospital for implantation of subcutaneous implantable cardioverter defibrillator (S-ICD). Screening of the body surface using an ECG screening tool determined eligibility for S-ICD. The two-incision technique was selected from a cosmetic point of view that wounding of young patients should be less. The implantation was performed in an electrophysiology laboratory under general anesthesia. Extra-thoracic lead of S-ICD was placed using a two-incision technique. The sleeve of the xiphoid incision was fixed firmly with ETHIBOND EXCEL® polyester suture. The position of the defibrillation lead was confirmed by chest X-ray (Fig. 2A). The patient was discharged on day 7 post-implantation. Telemedicine monitoring was performed via the LATITUDE ™ system. Although he was prohibited from exercising, he was unable to comply and was participating in baseball games the day following discharge.

Outpatient chest X-ray showed dislodgement of an extra-thoracic lead (Fig. 2B). The lead dropped out during the outpatient examination at 3 weeks post-implantation, and the lead tip was in the lower one third of the sternum. There was no over sensing in the pacemaker check when the lead dropped, and no inappropriate shock of the S-ICD was found. Immediately after re-hospitalization, the dropped S-ICD lead was removed the next day and re-placed by a three-incision technic under intravenous anesthesia (Fig. 2C).

**Figure 3:**
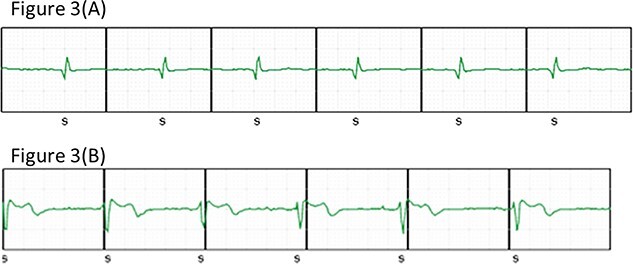
(A) Subcutaneous electrocardiogram (S-ECG) of remote monitoring SMARTPASS at pre-dislodgement, QRS positive of extrathoracic lead, and (B) S-ECG of remote monitoring SMARTPASS, QRS negative.

The alternative EGM in the remote monitoring SMARTPASS was later found to have changed the polarity of the QRS from positive to negative, and remote monitoring could detect the loss of the S-ICD lead (Fig. 3A and B).

## DISCUSSION

The two-incision technique is a safe and efficacious alternative for S-ICD implantations and offers physicians a less invasive and simplified implantation procedure of the S-ICD [5]. Lead dislodgement of S-ICD is a rare complication; however, there have been reports of leads dislodgement with the two-incision technique [6], due to patient motion or manual movement of the S-ICD generator [7]. We used the two-incision technique instead of three incisions for cosmetic reasons as the patient was a juvenile, but for patients that are sports players however, we use the three-incision technique in order to fix the lead more firmly. We found that the S-ICD lead may drop out in patients who perform much exercise as in the present case, so the three-incision technique is desirable and information by remote monitoring is useful.

## CONCLUSION

The three-incision technique may be preferable over the two-incision technique in the case of juveniles that are active in sports etc., and S-ICD patients.
